# Stimulation of P2X7 Enhances Whole Body Energy Metabolism in Mice

**DOI:** 10.3389/fncel.2019.00390

**Published:** 2019-08-21

**Authors:** Giacomo Giacovazzo, Paola Fabbrizio, Savina Apolloni, Roberto Coccurello, Cinzia Volonté

**Affiliations:** ^1^Preclinical Neuroscience, Fondazione Santa Lucia IRCCS, Rome, Italy; ^2^Institute for Complex System (ISC), CNR, Rome, Italy; ^3^Institute for Systems Analysis and Computer Science, CNR, Rome, Italy

**Keywords:** P2X7 receptor, BzATP, energy expenditure, oxygen consumption, fatty acid oxidation

## Abstract

The P2X7 receptor, a member of the ionotropic purinergic P2X family of extracellular ATP-gated receptors, exerts strong trophic effects when tonically activated in cells, in addition to cytotoxic effects after a sustained activation. Because of its widespread distribution, P2X7 regulates several cell- and tissue-specific physiological functions, and is involved in a number of disease conditions. A novel role has recently emerged for P2X7 in the regulation of glucose and energy metabolism. In previous work, we have demonstrated that genetic depletion, and to a lesser extent also pharmacological inhibition of P2X7, elicits a significant decrease of the whole body energy expenditure and an increase of the respiratory exchange ratio. In the present work, we have investigated the effects of P2X7 stimulation *in vivo* on the whole body energy metabolism. Adult mice were daily injected with the specific P2X7 agonist 2′(3′)-O-(4-Benzoylbenzoyl)adenosine 5′-triphosphate for 1 week and subjected to indirect calorimetric analysis for 48 h. We report that 2′(3′)-O-(4-Benzoylbenzoyl)adenosine 5′-triphosphate increases metabolic rate and O_2_ consumption, concomitantly decreasing respiratory rate and upregulating NADPH oxidase 2 in *gastrocnemius* and *tibialis anterior* muscles. Our results indicate a major impact on energy homeostasis and muscle metabolism by activation of P2X7.

## Introduction

The P2X7 receptor (Volonté et al., 2012; Di Virgilio et al., 2018), a member of the purinergic ionotropic P2X family of extracellular ATP-gated receptors, exerts a strong trophic effect when tonically activated in cells (Adinolfi, 2005), in addition to a well-recognized role in apoptotic cytotoxicity after a sustained activation (Di Virgilio et al., 2018). The receptor is present in cells of hematopoietic lineage including erythrocytes, monocytes, macrophages, dendritic cells, B and T lymphocytes, being also expressed by mast cells, fibroblasts, osteoblasts, osteoclasts and myocytes (Zhang et al., 2004). In the CNS, P2X7 receptors are described in astrocytes, microglia, oligodendrocytes, Schwann cells, as well as in neurons from olfactory and lateral septal nuclei, cerebral cortex, striatum, and hippocampus (Volonté et al., 2012). Because of such a widespread distribution, P2X7 regulates several different tissue-specific and cell-specific physiological functions, being also involved in a number of disease conditions (Cotrina and Nedergaard, 2009; Fumagalli et al., 2017).

In the last few years, a new role has emerged for P2X7 in the regulation of glucose metabolism and energy homeostasis. Heterologous expression of P2X7 in HEK293 cells for instance upregulates glycolytic enzymes and the glucose transporter Glut1, thus promoting adaptation to unfavorable extracellular conditions (Amoroso et al., 2012). In intestinal epithelial cells, the activation of P2X7 modulates glucose transport through the downregulation of the glucose transporter Glut2 (Bilodeau et al., 2014). In pancreatic islet, an increase in glucose concentration stimulates P2X7 transcription, thus suggesting that glucose exerts also a feedback signaling inside the cells by stimulating the expression of regulatory P2X7. On the other hand, loss of P2X7 function in mice results in changes of adipocyte distribution and lipid accumulation. In particular, a significantly increased body weight, epididymal fat pad mass, lipid accumulation in adipocytes, and/or ectopic aggregation in the form of lipid droplets are observed in genetically depleted P2X7 male mice, although in the absence of any significant difference in food consumption (Beaucage et al., 2014). Moreover, in response to high-fat/high-sucrose diets, mice lacking P2X7 exhibit severe and rapid hyperglycemia, glucose intolerance and impaired beta cell function. P2X7 levels are elevated in pancreatic beta cells of obese patients, but downregulated in patients with type-2 diabetes mellitus (Glas et al., 2009). The suppression of the CD36, a membrane glycoprotein modulator of lipid homeostasis and immune responses, playing critical roles in the uptake of fatty acids and involved in neurodevelopment, metabolic disorders, aging, dementia and stroke (Zhang et al., 2018) attenuates P2X7 and adipogenic protein expressions and decreases adipocyte differentiation (Gao et al., 2017). However, in another study, the activation of the inflammasome pathway in the adipose tissue was found associated with hyperglycemia and hyperinsulinemia but not mediated by the P2X7 signaling axis (Sun et al., 2012).

In a previous work, we have demonstrated that genetic depletion and, to a lesser extent, also pharmacological inhibition of P2X7 elicits a pronounced decrease of the whole body EE and a significant increase of the RER, thus indicating a prevalent increase of carbohydrate oxidation. The relative sparing of fatty acids storage and concomitant defective energy homeostasis were also associated to body weight gain (Giacovazzo et al., 2018). Given the increasing importance of P2X7 in the regulation of cellular energy metabolism, either during physiological or tumor conditions (Amoroso et al., 2012; Savio et al., 2018), in the present work we have investigated by IC the effects of P2X7 activation in mice that underwent 7-days treatment with the best available selective and specific P2X7 agonist BzATP. We demonstrate a significant increase of metabolic rate and O_2_ consumption, with a concomitant decrease of RER and increase in NOX2 expression in skeletal muscles, thus demonstrating a major impact on energy metabolism by activation of P2X7. Our results might contribute to shed further light on the role of P2X7 during metabolic diseases.

## Materials and Methods

### Animals

C57BL/6J mice were originally obtained from Charles River Laboratories (Lecco, IT) and maintained in the indoor animal facility. Animals were housed in groups of 4–5 mice/cage in standard conditions with free access to food and water, at constant temperature (22° ± 1°C) and relative humidity (50%), with a regular 12 h light cycle (light 7AM–7PM). All animal procedures were performed according to European Guidelines for the use of animals in research (86/609/CEE) and requirements of Italian laws (D. L. 26/2014). The Animal Welfare Office, Department of Public Health and Veterinary, Nutrition and Food Safety, General Management of Animal Care and Veterinary Drugs of Italian Ministry of Health have approved the ethical procedure. All efforts were made to minimize animal suffering and the number of animals necessary for obtaining reliable results.

### Pharmacological P2X7 Receptor Activation

Adult (15 weeks-old) C57BL/6J female mice were randomly grouped into vehicle- or treated-mice and daily i.p., administered, respectively, with vehicle PBS or the best available specific and selective P2X7 agonist BzATP (Sigma-Aldrich, Italy) used at 1 mg/Kg, (279.7 μM) for 7 days in standard feeding conditions. During the last 48 h (h) of treatment, mice were subjected to continuous IC recording.

### Energy Metabolism

Energy expenditure, volume of oxygen consumption (VO_2_) and RER were measured by an IC system (TSE Systems Manufacturer, model PhenoMaster/LabMaster^®^ System for Automated Home Cage Metabolic Phenotyping) with a constant air flow of 0.35 L/min, as described (McLean and Tobin, 1988; Grobe, 2017). Mice were adapted for 24 h to the metabolic chamber prior to recording, and VO_2_ and VCO_2_ were measured every 20 min, for a total of 48 h (12 h dark-light phase comparison). Room temperature and humidity were kept constant (22° ± 1°C).

As index of substrate oxidation, we used the ratio between the volume of CO2 (VCO2) produced, and the volume of O2 (VO2) consumed (RER = VCO2/VO2). EE was then calculated according to the equation VO2 × (3.815 + (1.232 × RER)), as provided by the TSE manufacturer. In particular, EE is calculated in terms of CV that is the relationship between the heat and VO2, as reported in Graham (1917). The resulting equation for the CV is 3.815 + 1.232 ^∗^ RER, and the heat is CV × VO_2__*subject*_, which is the rate of O2 consumed by each subject. Finally, the heat has been normalized using the estimated lean mass per mice, to express values as kcal/h/Kg.

The EE and RER for each of the sample points were evaluated across the 48 h of total recording. Locomotor activity was assessed during the indirect calorimetric assay by the number of infrared beams broken. Each cage of the calorimeter system is equipped with the InfraMot^®^ device that uses “passive infrared sensors” to detect and record the motor activity of the mouse by the body-heat image and its spatial displacement across time. EE was also analyzed by considering animals’ steady conditions or lack of motor activity (only values included between 0 and 3 activity counts were included) and indicated as resting EE (REE).

### Time Course of Consecutive BzATP, A804598 and A804598 Plus BzATP Administrations in Mice

After adapting to the metabolic chambers for 24 h, adult (15 weeks-old) C57BL/6J female mice (*n* = 4) were i.p. injected with 1 mg/Kg BzATP and energy metabolism recorded for 24 h (day 1), then i.p. injected with 90 mg/Kg A804598 and energy metabolism recorded for additional 24 h (day 2), and finally (day 3) administered with A804598 followed (20 min after) by BzATP and energy metabolism recorded for the last 24 h. Results are expressed as VO_2_ or EE (Kcal/hour/Kg).

### Western Blotting

Total protein extracts from mice *gastrocnemius* and *tibialis anterior* muscles were obtained in homogenization buffer (15 mg of dry tissue/150 μl of 20 mM HEPES, pH 7.4, 100 mM NaCl, 1% Triton X-100, 10 mM EDTA) added with protease inhibitor cocktail (Sigma Aldrich). After sonication and centrifugation at 14000 × g (20 min at 4°C) supernatants were collected and assayed for protein content by Bradford detection kit (Bio-Rad Laboratories, Hercules, United States). Protein separation and analysis (15 μg/well) was performed by Mini-PROTEAN^®^ TGX^TM^ Gels (BioRad, United States) and by transfer onto nitrocellulose membranes. After saturation with 5% non-fat dry milk (1 h at room temperature), membranes were probed with gp91phox antibody (1:1000, BD Transduction Laboratories, United States) in 5% non-fat dry milk overnight at 4°C, and incubated with HRP-conjugated secondary antibody (mouse 1:5000, Jackson Immunoresearch) for 1 h at room temperature. Detections were performed on X-ray film (Aurogene, United States), using ECL Advance detection kit (Amersham Biosciences, United States) and signal intensity visualized by Kodak Image Station and analyzed by ImageJ software (NIH, United States). Values were normalized with mouse anti-GAPDH (1:2500, Sigma-Aldrich, Italy).

### Statistical Analysis

Data are expressed as means ± standard error of the means. Statistical analysis was performed by Student’s *t-*test, or 1-way analysis of variance (ANOVA) followed by Tukey *post hoc* test. The accepted level of significance was set at ^∗^*p* < 0.05.

## Results and Discussion

**FIGURE 1:**
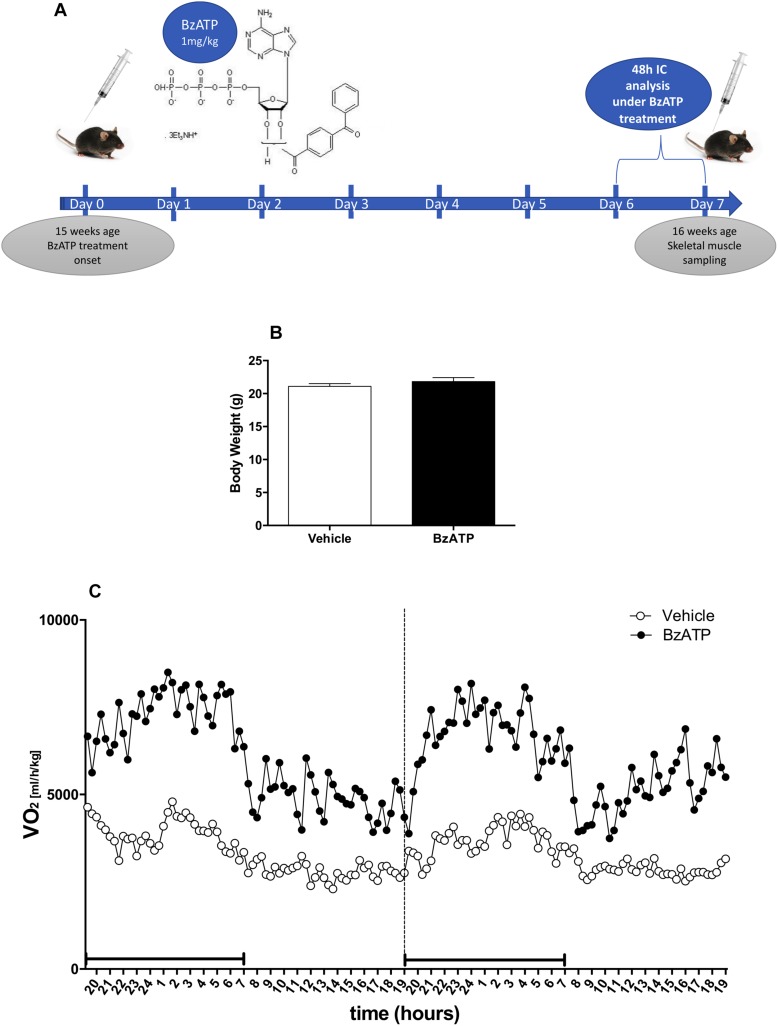
Experimental plan and effects of 7-days BzATP administration on whole oxygen consumption. Adult (15 weeks-old) female C57BL/6J mice were daily i.p., administered with PBS or 1 mg/Kg BzATP for 7 days. At day 5, vehicle (*n* = 5) and BzATP-treated mice (*n* = 5), were introduced in the metabolic chambers to adapt for 24 h, and then subjected to IC recording for 48 h (days 6 and 7). **(A)** Schematic representation of experimental plan and execution. **(B)** Mean (± SEM) body weight (g) collected during the IC analysis for 48 h. **(C)** Mean (± SEM) of 48 h metabolic activity expressed as volume of oxygen consumption rate (VO_2_) (ml/h/Kg), during the entire light/dark cycle. Black line below the recording time (*X*-axis) indicates the dark phase.

To investigate the role of P2X7 activation in the regulation of energy homeostasis, we have assessed the metabolic rate and the respiratory quotient of C57BL/6J mice treated once a day for 7 consecutive days with vehicle (PBS) or the best selective and specific available P2X7 agonist BzATP, by means of continuous IC analysis during the last 48 h of treatment in standard nutritional conditions (Figure 1A). To overcome sex-mixed results with low reproducibility, we have used only the female gender because of previously reported data (Giacovazzo et al., 2018). Our results indicate that BzATP at the concentration of 1 mg/Kg (i.e., within a range demonstrated to be effective in rodents (Gubert et al., 2016), shows quick absorption without accumulation in the peritoneal space. Moreover, BzATP is well tolerated presenting no signs of toxicity and apparent distress such as sunken flanks, neglected grooming, or piloerection, as evaluated by daily inspection of body appearance and behavioral parameters comprising locomotor activity. Mean body weight collected before BzATP treatment, and immediately before and after the IC analysis for 48 h, shows no differences with respect to untreated mice (Figure 1B). Moreover, the liver weight remains constant during the entire period of BzATP treatment (vehicle-treated mice 3,57 ± 0,02 gr/100 gr body weight, *n* = 2; BzATP-treated mice 3,89 ± 0,21 gr/100 gr of body weight, *n* = 4, not statistically significant difference).

**FIGURE 2:**
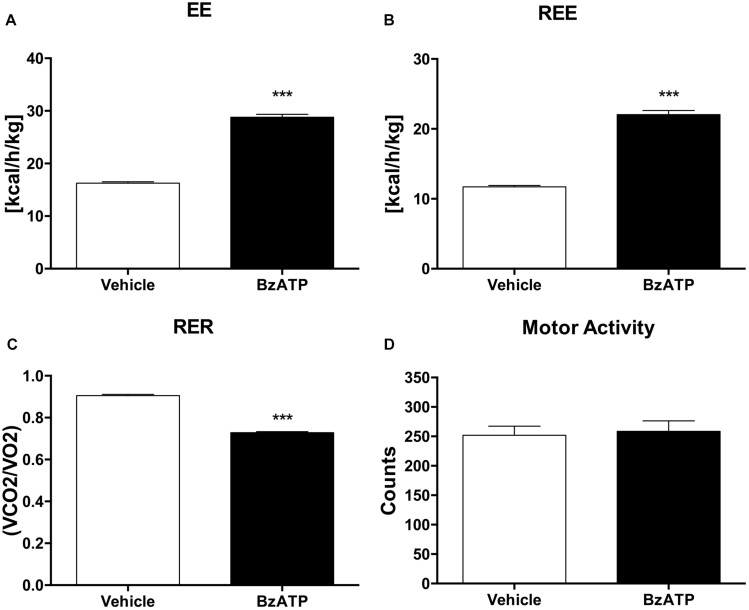
BzATP increases energy expenditure and reduces respiratory exchange ratio. **(A)** Mean (±SEM) of 48 h EE expressed as Kcal emitted by whole body per hour, per Kg of body weight (Kcal/h/kg). **(B)** Mean of 48 h resting EE (REE) expressed as whole body EE in lack of motor activity (Kcal/h/Kg). **(C)** Mean (±SEM) of 48 h respiratory exchange ratio (RER), expressed as ratio of produced CO2 volume to used O2 volume (VCO_2_/O_2_). **(D)** Mean (± SEM) of 48 h locomotor activity (counts). (^∗∗∗^*P* < 0,0001 vs. WT).

Having established that BzATP at the used dosage demonstrates no evidence of toxic effects, and that BzATP-treated mice present no gross pathology on necropsy compared to saline-treated mice, we have then assessed the metabolic rate, expressed as continuous volume of oxygen consumption, demonstrating a marked increase of VO_2_ intake across the 48 h period (Figure 1C). The *in vivo* IC analysis of EE demonstrates that the agonism at the P2X7 receptor produced a significant increase of metabolic rate (Kcal/h/kg) in terms of enhanced heat production and total EE (Figure 2A). Notably, the increase of EE is generated also in the lack of motor activity as detected during the overall resting period recorded across the entire light-dark cycle (Figure 2B). Moreover, the enhancement of EE is not attributable to significant changes of food intake (data not shown) and, consequently, to the contribution of food-induced thermogenesis. Next, we have collected data on the RER, in order to evaluate the differential level of nutrient substrate oxidation or, in other words, which energy source among carbohydrates, lipids or proteins is predominantly oxidized. Because the RER diminishes (Figure 2C) and more O_2_ is consumed (Figure 1C), our results indicate an increase of fatty acid oxidation and a prevalent use of lipid storage as energy source after P2X7 receptor stimulation. Of note, these changes are detected with no evident alteration of motor behavior (Figure 2D).

**FIGURE 3:**
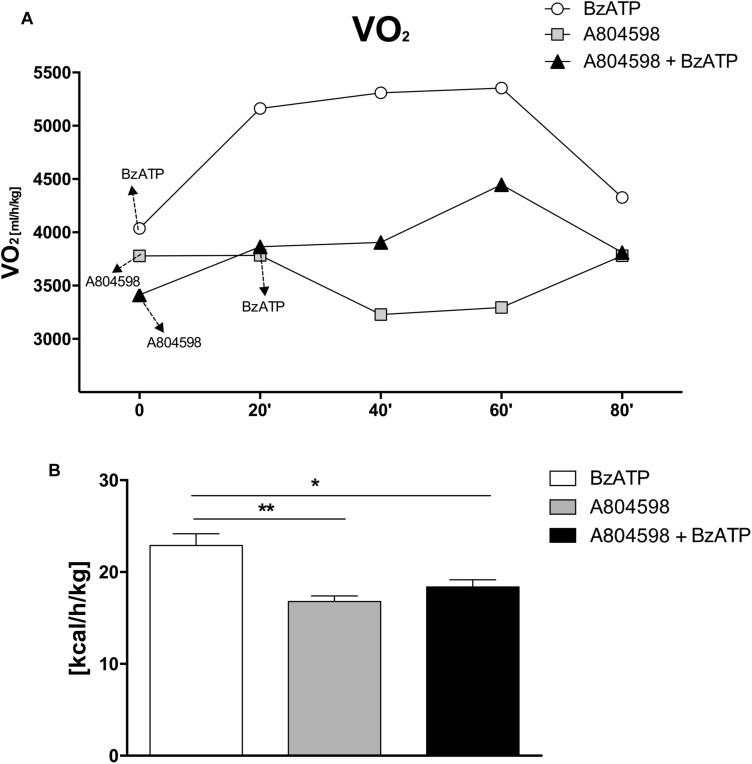
A804598 administration prevents the BzATP-dependent increase of energy expenditure. Female C57BL/6J mice (*n* = 4) after adapting to the metabolic chambers for 24 h, were subjected to IC recording for 3 consecutive days according to the subsequent experimental design: day1, a single BzATP 1 mg/Kg i.p. injection and IC recording for 24 h,; day 2, a single A804598 90 mg/Kg i.p. injection and IC recording for 24 h; day 3, A804598 injection followed (after 20 min) by BzATP and IC recording for 24 h. **(A)** Mean (± SEM) 24 h metabolic activity is expressed as volume of oxygen consumption rate (VO_2_) (ml/h/kg). **(B)** Mean (± SEM) EE energy expenditure is expressed as Kcal/h/Kg. (^∗∗^*P* < 0,001 BzATP vs. A804598, ^∗^*P* < 0,005 BzATP vs. A804598 + BzATP).

Altogether, these results clearly show that during the entire circadian period and under standard nutritional conditions, both EE and REE are significantly increased by BzATP, while RER levels are decreased, thus demonstrating a major activation of energy metabolism induced by the stimulation of P2X7. These findings complement our recent evidence that both transgenic loss and pharmacological impairment of P2X7 function conversely cause a robust decrease of metabolic rate and a lower ratio of fat to carbohydrate oxidation (Giacovazzo et al., 2018), thus generating lipid accumulation, increased fat mass distribution and, ultimately, the weight gain that is reported in P2X7-depleted mice (Beaucage et al., 2014). To further confirm the involvement of P2X7 in such events, we performed continuous IC recordings of C57BL/6J mice for 4 days, by registering basal activity for 24 h, followed by a single 1 mg/Kg BzATP i.p. administration and recording for 24 h, followed by a single i.p. administration of the specific P2X7 antagonist A804598 (90 mg/Kg, 24 h recording), and lastly BzATP (20 min pre-injection) followed by A804598 i.p. injection and final recording for 24 h. We demonstrate that the pre-administration of A804598 delays and attenuates the increase in VO_2_ consumption (Figure 3A) and EE (Figure 3B) induced by BzATP alone. These results further corroborate the previously observed implication of P2X7 in the decrease of whole body EE and increase of RER sustained by either pharmacological inhibition with the A804598 antagonist or genetic depletion of P2X7 (Giacovazzo et al., 2018).

**FIGURE 4:**
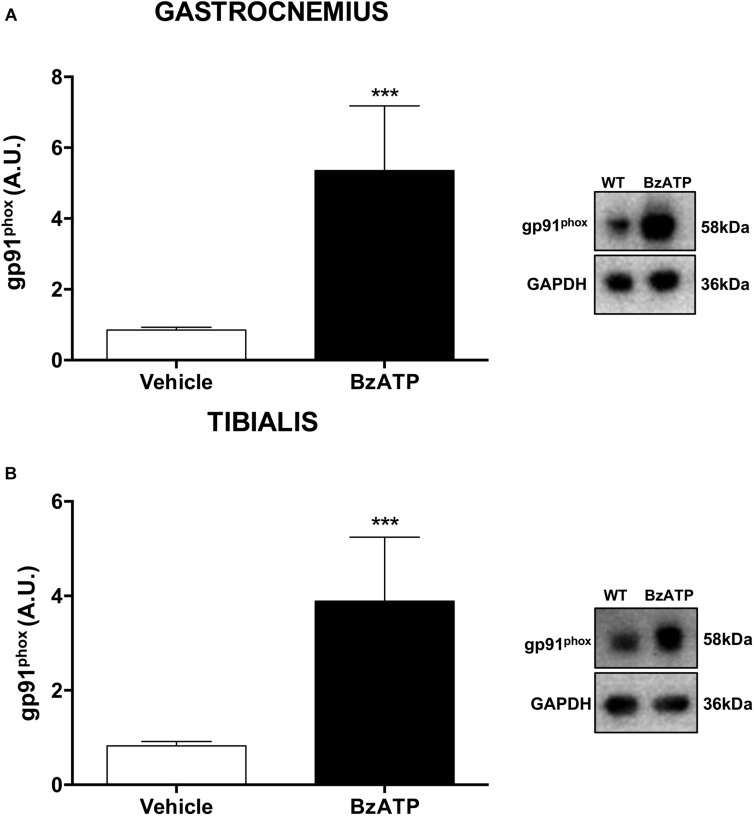
BzATP enhances gp91phox expression in skeletal muscles of C57BL/6J mice. An equal amount of total *gastrocnemius* **(A)** and *tibialis anterior* **(B)** muscle lysates from vehicle and BzATP- treated mice (*n* = 3/group) were subjected to SDS-PAGE and Western Blotting with anti-gp91^*phox*^. GAPDH was used for protein normalization. Data represent means ± SEM. Statistical significance was calculated by Student’s *t*-test, as referred to vehicle, ^∗^*p* < 0.05.

As one of the largest tissue accounting for about 50% of body mass, the skeletal muscle is a major determinant of the whole-body metabolic rate (Zurlo et al., 1990), possessing a remarkable capacity to rapidly shift from carbohydrates to fatty acids utilization in response to increasing energy request and intensity of physical exercise (Kiens et al., 1993; Romijn et al., 1993; Van Loon et al., 2001). Indeed, the skeletal muscle readily responds to changing metabolic needs due not only to physiological, but also pathological stimuli, and the redox homeostasis appears to be a central modulator of muscle plasticity (Lambeth, 2004). Generation of reactive oxygen species (ROS) is for instance augmented in skeletal muscles as part of a physiological response to exercise, adaptation to increased workload and optimization of the contractile capacity (Forman et al., 2014). There is strong evidence that NOX2, a super-oxide generating enzyme and major source of ROS under resting and contractile muscle conditions (Pearson et al., 2014), with its membrane-bound catalytic gp91phox and cytosolic regulatory p47phox subunits, can be accompanied by a change in mitochondrial content that is responsible for alterations in substrate utilization and whole body energy metabolism. Physical exercise is known to specifically increase NOX2 mRNA levels in the *gastrocnemius* (Loureiro et al., 2016). To investigate the potential involvement of NOX2 in regulating energy homeostasis by BzATP, we have next analyzed the expression profile of the glycoprotein gp91phox in the *gastrocnemius* (possessing both fast- and slow-twitch fibers) and *tibialis anterior* (with 95% fast-twitch fibers) muscles. Our results indicate that the content of gp91phox protein in both fiber types is remarkably increased in mice treated for 7 days with BzATP (Figures 4A,B). Because during muscle contraction there is a larger cytosolic ROS production with only a discrete mitochondrial signal (Sakellariou et al., 2012), the increased gp91phox expression may thus suggest that P2X7 activation plays a role in the contraction-induced intracellular signaling mediated by non-mitochondrial ROS sources such as NOX2. Notably, P2X7 activation by BzATP might also contribute to maintain a redox balance by inducing a physiological and transient ROS generation (Ristow, 2014; Merry and Ristow, 2016). In this view, the BzATP-induced activation of P2X7 appears to mimic the effects produced by high-intensity training and, particularly, the increase of oxygen consumption (VO_2_) (Trapp et al., 2008; Hazell et al., 2012) and the NOX2-dependent redox adaptation to endurance exercise that occur in both mixed (i.e., *gastrocnemius*) and fast-twitch glycolytic fiber types (i.e*., tibialis anterior*) (Henríquez-Olguín et al., 2019).

Overall, our results disclose and support a previously hypothesized function for P2X7 in shaping the whole-body energy metabolism (Giacovazzo et al., 2018). Here, we have shown that healthy mice challenged by BzATP display higher EE and higher oxidative capacity, with increased NOX2 expression in both mixed fast/slow-twitch and fast-twitch fibers. Because an increased oxidation of fatty acids reduces the carbohydrate utilization thus sparing the glycogen stores, and suppresses lactate production leading to increased endurance performance (San-Millán and Brooks, 2018), we suggest that a sustained activation of P2X7 might cause the remodeling of fatigue-resistance muscles and the reprogramming of the global muscle gene expression toward a slower, more oxidative transcription program.

Aging is associated with changing of muscle metabolism, adaptations in slow and fast fibers as well as a progressive decline of whole-body resting metabolic rate (Elia et al., 2000; Petersen et al., 2015). Altogether, muscle aging is associated to a drastic remodeling of muscle metabolism (e.g., decreased insulin sensitivity and mitochondrial capacity), structure (e.g., reduction of myofibers and muscle strength) and function (e.g., autophagy and contractile capacity) (Collino et al., 2013). Of note with aging, several glycolytic enzymes become overexpressed in the slow fibers, and a parallel upregulation and downregulation of glycogen metabolism is observed in slow and fast fibers of aged people, respectively (Rakus et al., 2015; Murgia et al., 2017). Since aging is also associated to muscle fiber type conversion, entailing a preferential loss of glycolytic fast-twitch fibers (Larsson, 1983), there is also the possibility that abnormal upregulation of glycogen metabolism in the slow fibers may, at least in part, accelerate the transition between fast- and slow-twitch fibers. In the light of our results, we might suggest that the potentiation of P2X7 function in adult mice might contribute to restore a proper balance in either aged or pathologic carbohydrate-driven energy metabolism, by forcing the balance toward a prevalent oxidation of fatty acids and enhancement of metabolic rate. Further experiments on the role of P2X7 function will provide important insight about the fine-tuning of energy metabolism and redox homeostasis within the skeletal muscle cell.

## Data Availability

The datasets generated for this study are available on request to the corresponding author.

## Ethics Statement

The animal study was reviewed and approved by the Animal Welfare Office, Department of Public Health and Veterinary, Nutrition and Food Safety and General Management of Animal Care and Veterinary Drugs of the Italian Ministry of Health.

## Author Contributions

RC and CV contributed to the conception and design of the study. GG, PF, and SA contributed to the data acquisition and data analysis. All authors have approved the final version of the manuscript.

## Conflict of Interest Statement

The authors declare that the research was conducted in the absence of any commercial or financial relationships that could be construed as a potential conflict of interest.

## Abbreviations

BzATP: 2 ′ (3 ′ )-O-(4-benzoylbenzoyl)adenosine 5 ′ -triphosphate
CD36: cluster of differentiation 36
CV: caloric value
EE: energy expenditure
h: hours
IC: indirect calorimetry
i.p.: intraperitoneal
NOX2: NADPH oxidase 2
PBS: phosphate buffered saline
RER: respiratory exchange ratio
VCO_2_: volume of CO_2_ production
VO_2_: volume oxygen consumption.
